# Development of a Smartphone Application to Measure Physical Activity Using Sensor-Assisted Self-Report

**DOI:** 10.3389/fpubh.2014.00012

**Published:** 2014-02-28

**Authors:** Genevieve Fridlund Dunton, Eldin Dzubur, Keito Kawabata, Brenda Yanez, Bin Bo, Stephen Intille

**Affiliations:** ^1^Department of Preventive Medicine, University of Southern California, Los Angeles, CA, USA; ^2^Department of Psychology, University of Southern California, Los Angeles, CA, USA; ^3^College of Computer and Information Science, Northeastern University, Boston, MA, USA; ^4^Bouvé College of Health Sciences, Northeastern University, Boston, MA, USA

**Keywords:** context-sensitive ecological momentary assessment, experience sampling, smartphone, mobile phone, physical activity, sedentary behavior

## Abstract

**Introduction:** Despite the known advantages of objective physical activity monitors (e.g., accelerometers), these devices have high rates of non-wear, which leads to missing data. Objective activity monitors are also unable to capture valuable contextual information about behavior. Adolescents recruited into physical activity surveillance and intervention studies will increasingly have smartphones, which are miniature computers with built-in motion sensors.

**Methods:** This paper describes the design and development of a smartphone application (“app”) called Mobile Teen that combines objective and self-report assessment strategies through (1) sensor-informed context-sensitive ecological momentary assessment (CS-EMA) and (2) sensor-assisted end-of-day recall.

**Results:** The Mobile Teen app uses the mobile phone’s built-in motion sensor to automatically detect likely bouts of phone non-wear, sedentary behavior, and physical activity. The app then uses transitions between these inferred states to trigger CS-EMA self-report surveys measuring the type, purpose, and context of activity in real-time. The end of the day recall component of the Mobile Teen app allows users to interactively review and label their own physical activity data each evening using visual cues from automatically detected major activity transitions from the phone’s built-in motion sensors. Major activity transitions are identified by the app, which cues the user to label that “chunk,” or period, of time using activity categories.

**Conclusion:** Sensor-driven CS-EMA and end-of-day recall smartphone apps can be used to augment physical activity data collected by objective activity monitors, filling in gaps during non-wear bouts and providing additional real-time data on environmental, social, and emotional correlates of behavior. Smartphone apps such as these have potential for affordable deployment in large-scale epidemiological and intervention studies.

## Introduction

One of the most significant continuing challenges in the physical activity field is the need for valid and reliable measures of physical activity and sedentary behavior in adolescents for surveillance, epidemiological, and intervention studies. Concern over the validity of retrospective self-report due to recall errors and biases, especially for youth (1–3), has led to an increase in the use of “objective” measures of physical activity and sedentary behavior, such as accelerometer-based activity monitors, heart rate monitors, and global positioning system (GPS) devices. For instance, a number of studies have found differences in physical activity levels and patterns when comparing self-report and objective (e.g., accelerometers and GPS) assessment methods (4–6). Currently, objective activity monitors are being deployed in large-scale surveillance studies with adolescents (7, 8) and offer a promising opportunity to obtain more accurate assessment of physical activity and sedentary behavior in this age group.

Objective monitors, however, often yield missing, incomplete, or unexplainable data that add complexity and cost to data cleaning and data analysis. Data may be incomplete for a variety of reasons, among them (1) participants forget to wear or carry monitors, (2) participants remove monitors when they do not want to, or cannot, wear them, (3) participants remove monitors during sleep, bathing, and swimming, and (4) device limitations such as low battery life, signal interference, and malfunction. Studies using accelerometers in children and adolescents typically have only about 50% of participants with seven or more complete days of data (9, 10) and 60–80% with four or more complete days (7, 11). Missing data are even more common with GPS monitors, which encounter signal drop inside some buildings or dense urban areas and battery drain after 24 h of use (12, 13). GPS data availability ranges from 11 to 60% of all possible records in recent studies (14–16) with user error as a significant cause of missing data in adolescents (17). A particular concern is that these data are not missing at random (e.g., Actigraph removed during a soccer game), which can result in biased activity estimates from objective monitors.

Objective activity monitors are also unable to capture valuable contextual information about physical activity and sedentary behavior such as activity type and purpose, mood, and social and physical milieu. According to the multilevel ecological framework, interactions between individual, social, physical, and environmental factors in settings in which people live, work, and play are important for predicting physical activity and sedentary behavior (18). A growing body of research suggests that concurrent physical and social contextual exposures (19–22) as well as affective, physical feeling, and motivational states (23) play an important role in determining levels of physical activity and sedentary behavior at any given moment.

Adolescents recruited into objective physical activity and sedentary behavior monitoring studies will increasingly have “smartphones,” which are mobile phones with built-in sensors and substantial computing power. Sophisticated programs can be easily installed on the phones (i.e., “apps”). The phones are rarely far from the adolescents, and adolescent affinity for the phones creates new opportunities for activity monitoring in surveillance and intervention studies. Mobile phones are being adopted throughout the U.S. population, including among lower-socioeconomic groups and minority populations (24); these phones expand options for health behavior measurement (25), and phone apps could be used to *supplement* existing data collection methods. In tandem with the peak of the smartphone industry, heightened consumer interest in physical activity measurement has resulted in dozens of devices and hundreds of applications designed to assist individuals in recording their day-to-day activities. While these devices and applications can track objective and subjective reports of physical activity independently, no known application has utilized accelerometry to assist individuals in labeling their day-to-day activities by prompting context-specific questions and showing a visual representation that aids in clustering activity throughout the day using phone motion. The current paper describes the design and development of a smartphone app that seeks to address the limitations listed above by combining objective and self-report assessment strategies to measure physical activity and sedentary behavior using sensor-informed real-time self-report and end of the day recall on typically carried mobile phones. This method utilizes data-driven participant self-report aimed at filling in gaps in objective activity data that result from device non-wear and malfunction. This method also supports the capture of time-sensitive contextual information about physical activity and sedentary behavior episodes that is not available from objective sensors.

## Methods

Mobile Teen is a new software technology (“app”) for smartphones that can automatically detect and elicit information about activity and data loss episodes through real-time sensor-informed context-sensitive ecological momentary assessment (CS-EMA) or experience sampling (26) and sensor-assisted end-of-day recall. The Mobile Teen software has two novel features: (1) a component that uses the mobile device’s built-in sensors to detect major transitions in type of phone movement or location, after which real-time CS-EMA questions are triggered that collect information about inferred physical activity, sedentary behavior, and data loss episodes, and (2) a second component that allows adolescents to interactively label their own activity data at the end of the day using visual cues from the phone motion and motion transitions to aid in recall of the type, intensity, duration, and start/stop times of those activities. Server-side software also remotely collects data from the Mobile Teen app in real-time and provides researchers with a cost-efficient way to remotely monitor participants during data collection to check for missing data. Following development, the software was tested through alpha and beta testing phases to verify that the app met the requirements that guided its design, worked as expected, could be implemented with the same characteristics as programed, and satisfied the needs of the user. All data obtained by the application was programed to transfer daily from the smartphone to a secure file transfer protocol (SFTP) server. At the conclusion of testing, smartphones were reset to factory settings and local phone data (including personal data) were erased with the participant present to preserve confidentiality. All participants were fully informed of the information gathered by the application, the purpose of the study, and the data purging process; consent and assent was obtained from each participant.

### Software development

The Mobile Teen smartphone app was designed by an interdisciplinary team of researchers consisting of computer scientists, psychologists, epidemiologists, exercise scientists, graphic designers, and users. The app was designed for mobile phones running the Android operating system (OS). The Android OS permits continuous raw data collection and processing from the phone’s internal accelerometer; iOS, the OS used on Apple iPhone devices, currently does not; on newer iOS phones summary motion data is provided. The Mobile Teen software was written in Java and targets Android version 2.3.3–4.3, the versions available at the time of the research. The application will run on most Android phones but was tested most thoroughly on LG Nexus 4 model phones, the model used in the pilot testing described in this paper.

Several rounds of iterative technical development and field testing were conducted, starting with storyboarding and low-fidelity paper prototyping exercises (27). The graphic user interface for the surveys was built following a format successfully in use in several other studies. The end-of-day labeling interface was tested on paper with a convenience sample of people in the research group and colleagues, after which a prototype was implemented. Software components were then sequentially added and technical problems resolved. Members of the programing team carried smartphones with the app active for several months to gather data on phone movement and adjust the algorithms used to identify transitions between clusters of the phone’s motion, so they map onto transitions between actual bouts of physical activity, sedentary behavior, and missing phone sensor data. Phones were setup to audio prompt (i.e., beep) in real-time when transitions were identified to provide an intuitive sense of the algorithm performance when triggering CS-EMA prompting, and pilot testing was performed in the lab to ensure the system would detect major transitions even when the phone is carried in different configurations (i.e., in various pockets, bags). Short pilot tests consisted of asking individuals to walk at different speeds and sit and stand in various ways to study the motion signals gathered from the phone. Members of the development team also used the app daily to refine the end of the day activity “chunking” interface, simplifying the initial design somewhat in the process and adjusting parameters such as the minimal length of a bout that can be labeled. Initial design ideas aimed at making the end-of-day labeling somewhat like a video game were ultimately revised in favor of a simpler design, due to concerns that the game mechanics would unduly influence participants to enter labels simply to complete the game, versus entering the labels that best captured activity.

### Alpha testing

Alpha testing to evaluate internal user acceptance, or feedback concerning typical use of the application, was conducted by four members of the study research team (including two research assistants, one graduate student, and a high school student intern), who did not have a direct role in programing the software. Members of the team carried the smartphone during their daily lives across designated periods of time spanning up to several days each. While carrying the phone, they maintained detailed logs of the dates, exact times, and types of CS-EMA survey prompts that were received. Members of the team also reported technical problems experienced with the Mobile Teen app and provided the programmers with additional feedback in order to refine the prototype application.

### Beta testing

Limited beta testing with external users was done to assess acceptance, usability, and feasibility of the end of the day recall component of the app. A sample of six high school students enrolled in grades 9–12 (63% male) and living in the Los Angeles metropolitan area participated in this phase. Participants were asked to carry a LG Nexus 4 smartphone with the Mobile Teen app installed for one full day. At the same time, they were also asked to wear an Actigraph GT3X accelerometer on a waist belt. At the end of the 24-h period, a member of the research team guided participants through the completion of the end of the day recall. After completing this component, participants were interviewed to assess their experiences and satisfaction with the software. Sample interview questions included the following: (1) “Could you please explain to me what the Mobile Teen Game^1^ does in your own words?” (2) “Did you feel that the instructions were clear on how you start the game and choose what day to begin?” (3) “Did you feel that the instructions were clear on what each part of the user interface does?” (4) “Do you have any suggestions for changes in appearance that would help others better understand or play the game?” Participants were compensated $20 for completing this beta testing component. This research was reviewed and approved by the Institutional Review Board at the University of Southern California.

Out of the six adolescents participating in the beta testing, five were able to label their activities using the end of the day component of the app. Data for one participant were irretrievable due to an application crash during the 24-h wear period. Interview feedback suggested that the app needed a wider range of activity categories, more icons, additional empty space so that each participant could enter their own activity, and the ability to split the activity bout more precisely for labeling. The Mobile Teen software was further refined based on this feedback provided by beta testing.

## Results

The Mobile Teen app has two major components: (1) sensor-informed CS-EMA, and (2) end-of-day sensor-assisted recall.

### Sensor-informed CS-EMA component

The app uses the mobile phone’s built-in motion sensor to automatically detect periods of motion, inactivity, or no-data from the phone. The app then uses these sensor-informed movement transition cues to trigger real-time CS-EMA self-report surveys measuring the type and purpose of activity previously performed, enjoyment of that activity, and social and physical features of the activity setting. EMA is a measurement strategy to elicit real-time self-report responses to electronic surveys in naturalistic settings throughout the course of daily life (28–32). To date, EMA studies have provided useful insight into the role of physical activity determinants such as pain and fatigue (33–35), affective states (36–42), intentions and social support (43, 44), and social and physical contextual influences (20, 21, 45–48).

#### Activity bout detection

On LG Nexus 4 phones, pilot testing showed that the accelerometer could be monitored continuously and achieve waking-day performance on a single charge (but without extensive additional use of the phone). To increase battery life to allow for typical use of the phone and to increase software reliability on some models of Android phones, the app samples the accelerometer for 20 s each minute^2^. Pilot testing suggested that 20 s/min of monitoring is sufficient to represent activity across the entire minute, for the purpose of triggering questions during the day and labeling activities at the end of the day.

Accelerometer data are captured at 10 Hz. A 1-s summary value is computed by taking the sum of the absolute value of the derivative of the *x*, *y*, and *z* axes. This number approximates the overall amount of acceleration change and can be easily computed in real-time.

Periods of data are then classified into three categories: no-data, low-intensity data, and high-intensity movement. Periods of no-data are easily identified if the app finds any time spans with missing data because if the app is running, data are recorded. If the phone is turned off or the app shuts down for some reason, when the phone is restarted the app also restarts. Periods of high-intensity data are detected using a set of heuristics that determine if there is a large change in the summary values (i.e., high second derivative of the original signal) or a substantial change in the average summary values around a given point, computed over the previous and next minute (and limited by rules that ensure no more than one chunk is proposed every 2 min). The motion in a high-intensity chunk must be above a threshold that was experimentally determined to represent significant ambulatory motion. All other periods are labeled as low-intensity motion; this category includes periods where the phone is recording data but not moving. The detected transitions, bouts, and motion summary values are stored for later analysis, but they are also used by the software to trigger real-time CS-EMA prompting. The app is designed to detect major activity transitions regardless of body placement of the smartphone on the user (e.g., pocket, hand, purse, and bag). The goal is for the app to function in an environment in which the user carries the phone naturally. For example, even if the user places the phone on a nearby desk or table when sitting, it is likely that he or she will carry the phone when transitioning to another room to keep it accessible for texting, Internet use, and calling (49).

#### Triggering rules

The app is programed with three rules for triggering CS-EMA survey prompts based on the phone’s built-in motion and power sensors (see Table 1). These rules were developed to detect automatically the natural end of bouts of physical activity, sedentary behavior or device non-wear, and the device being powered off. The trigger indicators are based on the average activity intensity value computed across a moving window for the timeframes specified in the table. The software is fully customizable and flexible to accommodating different activity thresholds or moving time windows as may be desired in other studies on different populations or circumstances.

**Table 1:** **Sensor-informed context-sensitive ecological momentary assessment (CS-EMA) triggering rules**.

Type of trigger	Indicator
1. Physical activity bout	15+ min of high-intensity activity followed by 10+ min of low-intensity activity
2. Sedentary behavior bout or device non-wear	60+ min of low-intensity activity followed by 1+ min of moderate intensity activity or greater
3. Device powered off	10+ min of no activity data followed by 1+ min of some activity data

#### Sampling and procedures

The sampling timeframe can be tailored within the software to meet the researchers’ needs. Typically, adolescents are asked to carry the phone as usual (either in their pockets, hands, purses, or bags) during waking hours. CS-EMA prompts are triggered during 2-h windows set by the researchers, during non-school hours (3–9 p.m. on weekdays and 7 a.m. to 9 p.m. on weekend days). Upon receiving an auditory CS-EMA prompt (a pleasant but loud 4 s chime), participants are instructed to stop their current activity and complete a short electronic survey question sequence using the touch screen of the smartphone. This process usually requires about 2–3 min. If a CS-EMA prompt occurs during an incompatible activity (e.g., sleeping or bathing), participants are instructed to ignore it. If no entry is made, the phone emits up to two reminder prompts at 3-min intervals. After this point, the electronic CS-EMA survey becomes inaccessible until the next prompting opportunity. Signal or interval-contingent EMA prompts can also be programed within the app. Unlike the CS-EMA prompts, which are triggered automatically immediately after the activity and data triggers listed in Table 1, signal-contingent EMA prompts occur at random times throughout the day based on frequency and boundary rules customized within the program. Combining signal-contingent (“random”) EMA prompting schedules with CS-EMA prompting can provide within-person comparison (i.e., “control”) conditions (29, 50). For example, they may allow researchers to compare negative mood immediately after physical activity bouts (captured though the CS-EMA) with negative mood occurring at other randomly prompted times throughout the day (captured by signal-contingent EMA).

To avoid excessive prompting, the app enforces a 30-min gap between all prompts. Therefore, if a context-sensitive prompt is presented and there is <30 min to the next scheduled signal-contingent prompt, then the signal-contingent prompt will not be presented for that particular 2-h window of time.

#### Self-report items

The app is programed with an EMA question sequence that is designed to measure major activity types, smartphone placement on the body, reasons for smartphone non-wear, and other psychological and contextual factors related to behavior. These EMA question sequences can be tailored to the unique hypotheses of the researcher. The app contains features to accommodate item dependencies, branching and skip sequences, and programed item missingness patterns.

**Figure 1:**
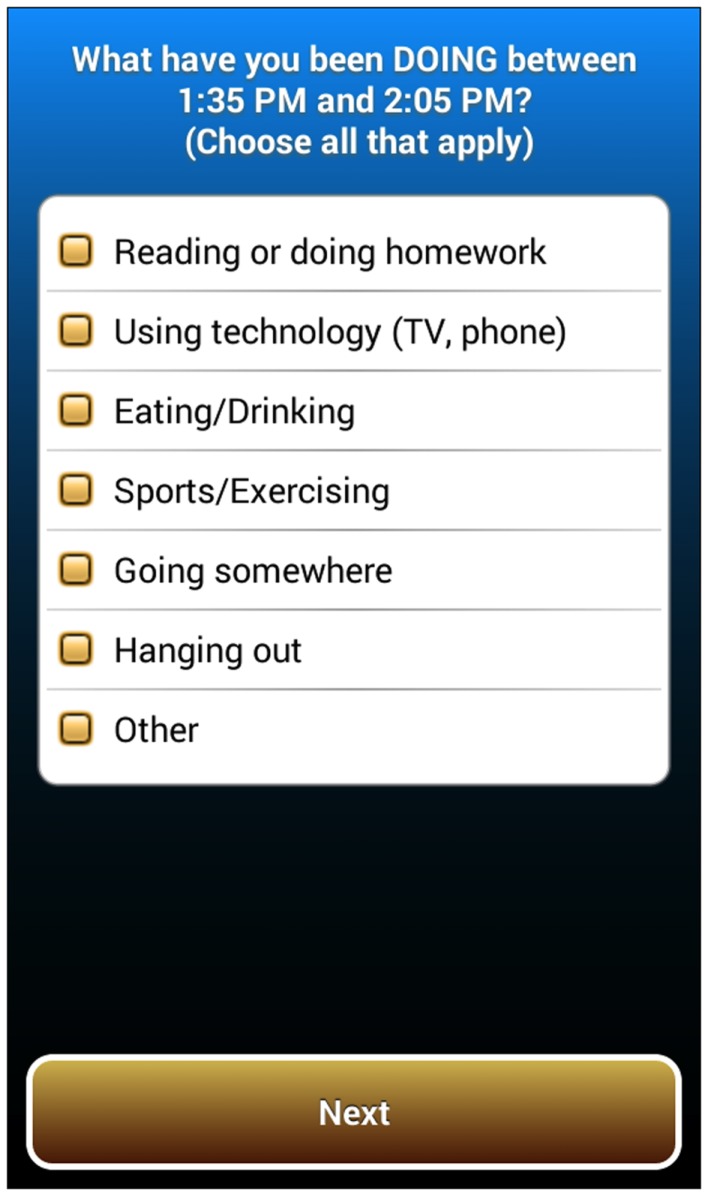
**Screenshot of the initial *activity type* question for the sensor-informed context-sensitive ecological momentary assessment (CS-EMA) component**.

The CS-EMA question sequence begins with a basic *activity type* question, “What did you do between (start time) and (stop time)?” where the start and stop times are automatically inserted by the app based on information from the built-in smartphone motion sensor and the particular triggering rule applied (see Figure 1). For example, if there was a Rule 2 trigger (60+ min of low-intensity activity followed by 2+ min of moderate intensity activity or greater), the first CS-EMA question would read, “What did you do between 10:33 and 11:48?” Alternatively, the signal-contingent (“random”) EMA question sequence begins with the *activity type* question, “What have you been doing for the past 30 min?” For both the CS-EMA and signal-contingent activity type questions, a response structure is used where participants may select multiple activities (i.e., “choose all that apply”) to indicate that they were multi-tasking. Response options include “*Reading or doing homework*; *Using technology* (*TV*, *phone*); *Eating/drinking*; *Sports/Exercising*; *Going somewhere*; *Hanging out*; *Other*.” This question is followed by a series of branched questions depending on the initial responses. For example, if *Sports/Exercising* is reported as an *activity type*, a follow-up question asks about the specific type of sports or exercise activity (e.g., *Basketball/Football/Soccer*, *Other running/Jogging*, *Exercise/Dance/Karate class*, *Weightlifting/Strength training*). Branching sequences for other *activity type* question responses are shown in Table 2.

**Table 2:** **Branching sequence for the activity type ecological momentary assessment (EMA) item**.

**INITIAL ITEM**								
**Item**	**Item wording**	**Response options (bold-face initiates first branch)**						

Activity type	What have you been doing between (start time) and (stop time)?	Reading or doing homework	**Using technology (TV/phone)**	Eating/drinking	Sports/exercising	**Going somewhere**	Hanging out	**Other**

**FIRST BRANCH SEQUENCE**								
**Item**	**Item wording**	**Response options (bold-face initiates second branch)**						

Using technology (TV, Phone)	While using technology (TV, phone), were you:	Playing video games	Talking	Texting	Using the internet	Watching TV/shows movies	Other	
Going somewhere	While going somewhere, were you:	Walking	Biking	Riding in a bus	Riding the metro/train	Riding in a car/taxi	Other (skateboarding, etc.)	
Other (1/2)	What was this other activity?	Doing chores/cooking	Showering/bathing	Sleeping	Working/part-time job	Getting ready for something	Shopping	Getting dressed
Other (2/2)	What was this other activity?	Class/school	Playing with children	Playing catch	Waiting	**Doing something else**		

**SECOND BRANCH SEQUENCE**								
**Item**	**Item wording**	**Response options**						

Doing something else		Write-in						

After indicating *activity type*, participants were asked, “Approximately how many minutes did you spend [answer from question about *activity type*]?” Next, participants are asked to report their body position (e.g., *Lying down*, *Sitting*, *Standing*) and how the phone was carried (e.g., *On my belt*, *In my pocket*, *Not with me*). If a participant indicates that the phone was not with him or her, the reason for not carrying the phone is asked (e.g., *Forgot it*, *Did not want to damage it*, *Too uncomfortable*). These questions about duration, phone placement, and reason for non-wear are repeated for each *activity type* initially reported and asked 100% of the time.

When “Sports/Exercising” is reported as an *activity type*, a branching sequence is triggered that asks also about what fitness skill was involved (e.g., *Flexibility*, *Strengthening*, *Endurance*), extra weight carried (e.g., None, <5, 5–10 lbs), degree of incline (e.g., *Mainly going uphill*, *Mainly going downhill*, *Mainly staying on flat ground*), perceived pain or soreness during that activity (e.g., *None*, *A little*, *Some*), and the physical context of that activity (e.g., *Home*, *Work*, *School*). Each of these questions is programed to randomly appear in only 40% of the *Sports/Exercising* follow-up question sequences to reduce participant response burden.

Additionally, for each *activity type* reported, a series of follow-up questions asks about the main purpose of the activity (e.g., *Fun/Recreation*; *To get somewhere*; *For work*, *homework*, *or housework*), how enjoyable it was (e.g., *Not at all*, *A little*, *Moderately*), intrinsic/extrinsic motivation for that activity (e.g., *You want to do it*, *Your* [*Parents*, *Friends*, or *Teachers*] *want you to do it*), and the social context of that activity (e.g., *Alone or With Friends*, *Parents*, *Siblings*). Each of these questions is programed to randomly appear in only 30% of the *activity type* follow-up question sequences.

### Sensor-assisted end-of-day recall component

**Figure 2:**
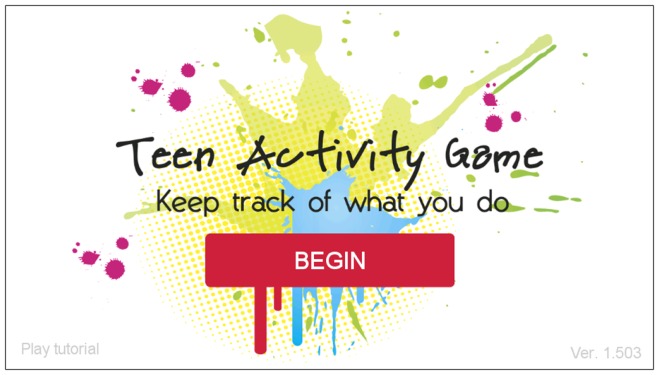
**Screenshot of the initial splash screen for the sensor-assisted end-of-day recall component**.

**Figure 3:**
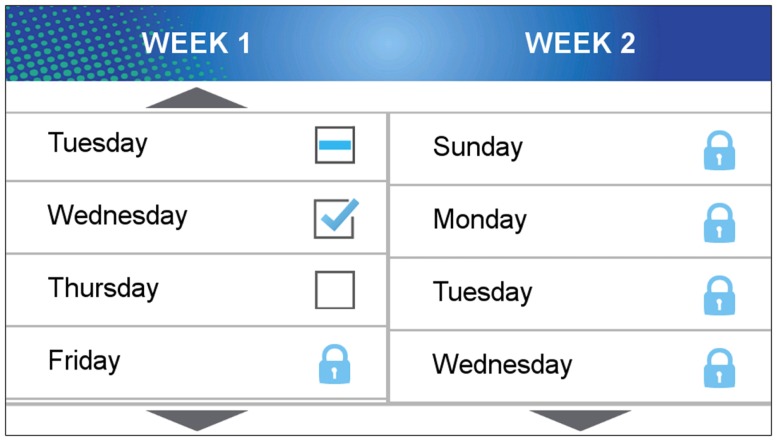
**Screenshot of the day selection screen for the sensor-assisted end-of-day recall component**.

The sensor-assisted end-of-day recall component allows adolescents to interactively label their own activity data each evening using the movement of their mobile phones to cue memory about the type, intensity, and duration of activities. Automatically detected bouts of activity, sedentary activity, or missing data provide activity start/stop boundaries. Participants are instructed to use the application each evening, or more frequently if they prefer, to label the activities for the previous 24 h. Upon launching the application, participants are presented with a horizontal splash screen with a “Begin” button and a “Play Tutorial” button (see Figure 2). The tutorial button guides participants through the end-of-day activity labeling procedure. After pressing the “Start Game” button, the app displays a selection of days for the given 2-week period, where each day has three possible status icons: expired and inaccessible (dash), complete and accessible (checkmark), incomplete and accessible (open box), or pending and inaccessible (lock symbol) (see Figure 3). Once a day begins, it is open and accessible for 48 h, after which it expires and can no longer be labeled. If the day is fully labeled, it is marked complete, but it can be accessed for corrections for up to 48 h. Pending days in the future are locked and inaccessible.

#### Activity “chunking”

**Figure 4:**
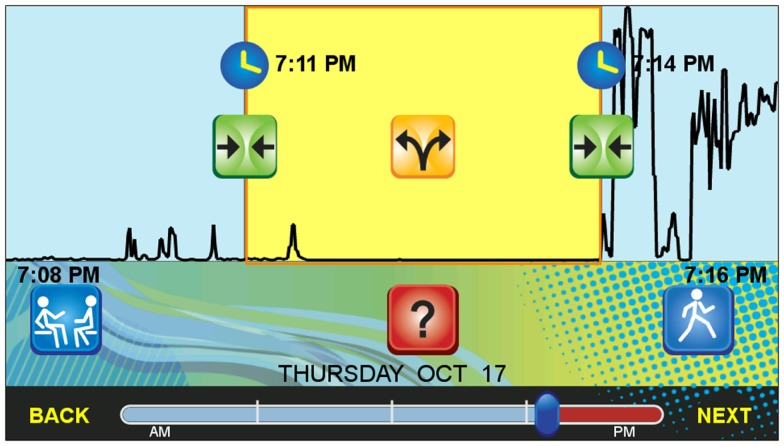
**Screenshot of the user interface for the sensor-assisted end-of-day recall**.

After the participant chooses a day, the app advances to a visual display screen for that day that is designed to assist the participant in recalling activities. The top half of the screen shows a line graph that represents the intensity of physical activity captured by the built-in accelerometer of the mobile phone (see Figure 4). Low and relatively flat lines indicate little phone motion (typically corresponding to sedentary behavior or not carrying the phone) and spikes, peaks, and elevated plateaus indicate substantial phone motion (typically corresponding to body movement). The vertical axis initially used dynamic scaling, but during pilot testing dynamic scaling was found to be confusing; the vertical axis is now fixed so that a typical walking motion with the phone in the pocket will result in the line being one third of the range. The absolute values of the vertical axis are not important as long as typical bouts of ambulation appear as clearly distinct in the graph from no or little movement and are roughly consistent across days. The horizontal axis on this graph is time as indicated by date and time stamps at the bottom of the screen (see Figure 4). A participant can navigate across the activity graph using inertia touch scrolling. Section “Activity Bout Detection” described the algorithm used to detect bouts of activity or missing data. The visual display indicates the beginning and end of each activity or missing data “chunk” (i.e., bout) with vertical lines (see Figure 4). By inserting hypothesized transition points based on the data, the app accomplishes three goals. First, it speeds up data entry when the bout start/stop times are detected accurately from the phone’s motion data. Second, if the bouts are not detected properly, having the transition points marked (but so they are easily moveable) will also save time. Finally, third, the application is gently suggesting to the user that certain time periods are sufficiently important to label. The activity “chunking” feature therefore both assists with identifying and recalling discrete activities, including the start/stop timing, and also can make the recall-based labeling task more efficient. Participants are asked to label their day in as much detail as possible, and to label each identified bout. When a bout is selected for labeling, clock face icons and time stamps appear on the vertical bout separation lines to indicate the beginning and end of the activity segment (see Figure 4). Also when selected, the bout changes to a yellow color and additional buttons appear that enable activity-frame manipulation.

#### Merging and splitting activity “chunks”

Green buttons with facing arrows, which sit beneath the clock face icons on the vertical lines, allow the participant to “merge” the selected activity bout with the adjacent bouts to the left or right if the activity contained within the highlighted bout is the same as prior or subsequent bouts (see Figure 4). Also, a yellow button with dividing arrows, which is positioned in the middle of the bout, allows the participant to “split” the segment into two equal bouts if two or more different activities were performed within the highlighted bout. A small number of taps therefore allow for efficient splitting, merging, and start/stop time adjustments for bouts. This is important because although the bout detection has been tuned based on the iterative pilot testing, no amount of tuning will lead to a perfect algorithm, and the algorithm often has insufficient information to split or merge certain types of bouts (e.g., a bout of *Eating/Drinking* that transitions without any extended ambulation to *Watching shows/Movies* could look like a single, extended bout based on phone motion).

Participants are told to merge activity bouts that were inappropriately split, and to split activity bouts that consist of more than one type of activity. A user, for example, might need to split a long bout of missing phone data or limited phone motion into different activities. The application allows bouts as short as 2 min.

#### Activity labeling

**Figure 5:**
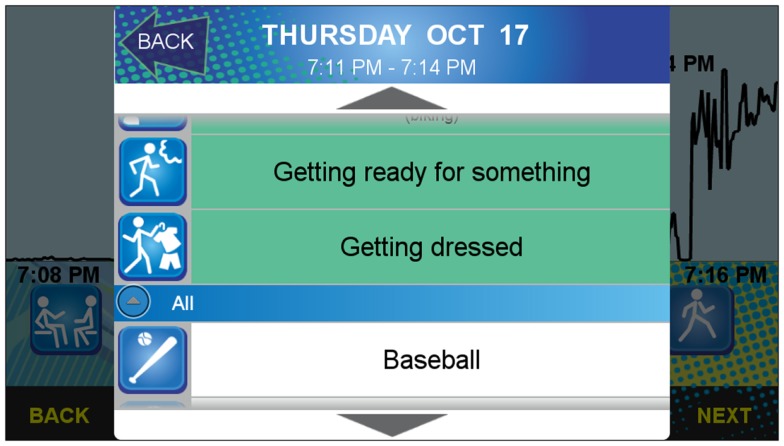
**Screenshot of the activity selection list from the sensor-assisted end-of-day recall component**.

**Figure 6:**
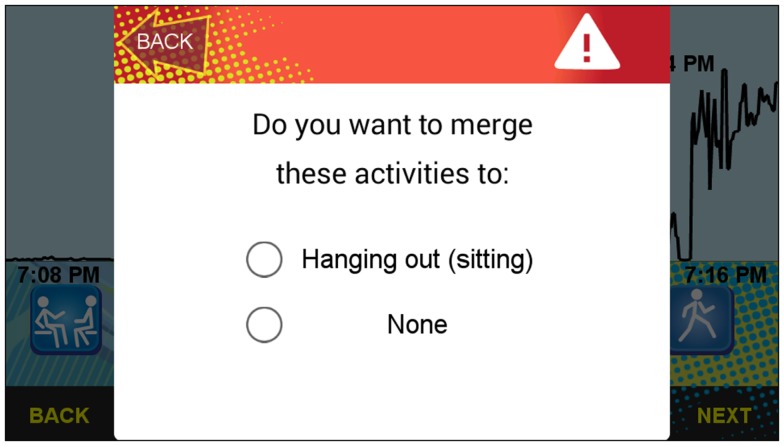
**Screenshot of the merging feature of the sensor-assisted end-of-day recall component**.

The center section of the screen functions as the main console for activity labeling. Unlabeled bouts are identified with an orange button with a question mark. After touching this question mark button, an activity selection list appears (see Figure 5). It contains a list of 46 common activities performed by adolescents (e.g., jogging, eating/drinking, sleeping) (see Table 3) adapted from the 3-Day Physical Activity Recall (3DPAR) (51) and Compendium of Physical Activities (52). Each activity has a corresponding visual icon, and the three most recently selected activity choices appear at the top of the list in green^3^. The remainder of the activity list is organized alphabetically. Once an activity label is selected for the highlighted bout, the orange question mark button is replaced with the respective activity icon. The participant may change his or her selection at any time by touching any existing icon and choosing another activity via the pop-up activity selection list. If a participant tries to merge two bouts that do not have matching labels, a warning pops up and requests further input to determine whether the new merged bout should contain one of the existing labels or remain unlabeled (see Figure 6).

**Table 3:** **Sensor-assisted end-of-day recall activity list**.

Activities				
Baseball	Basketball	Bicycling	Cooking/baking	Dance class
Doing chores	Eating/drinking	Fitness class	Football	Getting dressed
Getting ready for something	Going somewhere (biking)	Going somewhere (car/bus/train)	Going somewhere (skateboarding)	Going somewhere (walking)
Hanging out (sitting)	Hanging out (standing)	Jogging	Karate class	Other sports/exercise
Playing catch	Playing with child(ren)	Reading/doing homework	Running	Shopping food
Shopping other	Showering/bathing	Sitting in class	Skateboarding	Sleeping
Soccer	Swimming	Tennis/racquetball	Using computer/tablet	Using phone for anything (sitting)
Using phone for anything (standing)	Waiting (sitting)	Waiting (standing)	Walking	Watching shows/movies
Weightlifting/strength training	Working/job (sitting)	Working/job (standing/walking)	Doing something else (sitting)	Doing something else (standing)
Doing something else (walking)				

The bottom portion of the screen consists of a red bar quartered with white lines to delineate the day. As the participant advances through the labeling task, the bar changes from a red to a light blue color as a visual aid to indicate progress toward the completion of labeling (see Figure 4). A dark blue divider on the bar is used to represent the current visible time frame of the activity graph and functions as a navigation slider that can be moved to advance throughout the day. Additionally, movement between activity bouts is aided by “Back” and “Next” buttons at the bottom of the screen, which advance to the previous or next unlabeled bout, respectively. Periods of time in the future cannot be labeled. A participant who labels once per day in the evening begins labeling the evening of the prior day, completes that day, and then labels from midnight until the current time.

**Figure 7:**
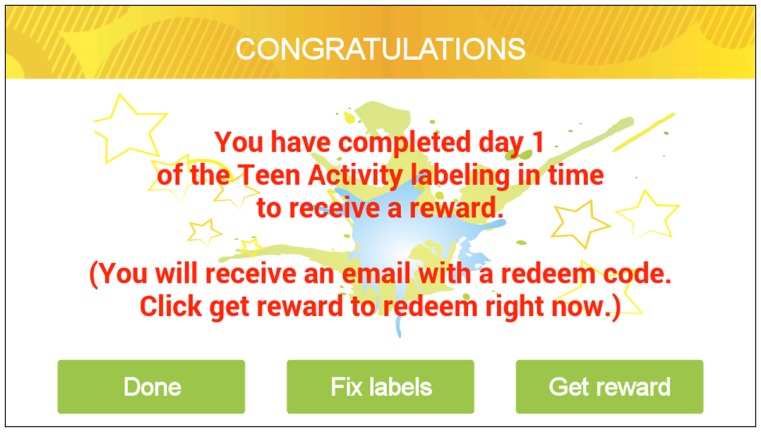
**Screenshot of the reward redemption at the end of the sensor-assisted end-of-day recall component**.

After all activity bouts are labeled for a particular day (i.e., a label is provided for the entire 24 h period) and the participant touches the “Next” button, the app advances to the reward splash screen (see Figure 7). The splash screen congratulates the participant for completing the respective day and allows the participant to exit with no action (Done button), fix labels in the previous day (Fix labels button), or obtain the unlocked reward for completing the labeling (Get reward button). The reward is distributed using an Amazon gift code, which can be immediately redeemed for $1 accessed through a redirect to the Amazon website on the phone. An email with the gift code is also sent to participants so that the participant can redeem it at a later time if preferred.

## Discussion

The self-reported activity information collected through the Mobile Teen app can be used to augment objective physical activity data collected by externally worn accelerometers or the smartphone’s built-in sensor. Data gathered by the app have the potential to enhance physical activity research and practice in a number of areas. First, these sensor-informed self-report data may significantly improve our understanding of objective activity device non-wear. Second, information about how the smartphone is carried (e.g., in my pocket, in my bag, or purse) from the CS-EMA can assist researchers in understanding the importance of smartphone body placement to activity assessment when using the phone’s built-in accelerometer. Third, the CS-EMA data can be used to adjust energy expenditure estimates for activities not well captured by waist-worn motion sensors (e.g., cycling, load-bearing, inclined). Fourth, sensor-informed CS-EMA and end-of-day recall data can also be used differentiate between conceptually distinct activity types (e.g., homework versus watching TV or soccer versus football) that may appear identical when examining objective activity intensity data alone. Fifth, contextual and psychosocial information collected by the CS-EMA component can be used to test hypotheses about real-time environmental, social, motivational, and emotional correlates of physical and sedentary activity. Each of these methodological benefits is described in further detail below.

### Improved understanding of objective activity device non-wear

Sensor-informed CS-EMA data from the Mobile Teen app running on a participant’s normal phone can allow researchers to more clearly and reliably differentiate between sedentary activity periods and true device non-wear for objective sensors used in research studies. Typically, researchers have defined device non-wear for Actigraph accelerometer (ACT)-based activity monitors by continuous periods of 0 activity counts for up to 60 min or more (53). However, there is some disagreement over the appropriate length of non-activity time (e.g., 20 and 60 min) to be used to define non-wear and whether short interruptions during that non-activity time reliably indicate device wear (54). Detecting non-wear of other types of activity monitors, such as GPS devices, relies on similar monitor-specific heuristics that estimate non-wear from data loss.

Most adolescents are highly motivated to carry and keep charged and operational their own personal phones. The accelerometer data from the phones will therefore capture major transitions throughout the day. Bouts of activity between transitions may correspond to bouts of non-wear of objective activity monitors, but data will still be gathered on these periods in time. This will permit identification of time periods when the objective monitor shows no-data but yet a participant reports meaningful activity, thereby confirming objective monitor non-wear.

Periods of phone non-wear are likely to correspond to periods of objective monitor non-wear. The Mobile Teen app CS-EMA trigger in response to Rule 2 (i.e., 60+ min of low-intensity activity followed by 2+ min of moderate intensity activity or greater) will ask participants how they carried the phone during the low-intensity time period detected by the app; the trigger in response to Rule 3 (i.e., 10+ min of missing phone data) will as well. If a participant responds with *Within reach*, *but not on me* or *Not with me*, then that particular period of time can be reasonably assumed to be phone device non-wear. The app will then ask about the reason for objective device non-wear (e.g., *forgot it, too uncomfortable*). The more information researchers have available about when and why adolescents are unwilling or unable to wear objective activity sensors, the more able they will be to adjust and improve research protocols to reduce overall non-wear rates. For example, if it turns out that a certain subgroup of adolescents tends to regularly forget to wear a research study objective sensing device, then researchers can devise methods to remind them such as triggering smartphone notifications, sending SMS messages, or enlisting parental assistance (55).

Lastly, self-reported *activity type* information from both the CS-EMA and the end of the day recall components can be used to estimate energy expenditure during device non-wear periods such as while swimming and participation in high-contact sports; most motion sensors are not waterproof and are often prohibited in team sports that involve collision. Activity categories selected through CS-EMA or the end of the day recall to report what the participant did during non-wear periods can be converted to metabolic equivalents (METs) using the Compendium of Physical Activities (52) and multiplied by the duration of known device non-wear (in minutes) to generate an estimate of energy expenditure (in MET minutes) for that period of time. These energy expenditure estimates can then be imputed to fill non-wear holes in objective activity data to obtain a more accurate representation of levels of physical activity and sedentary behavior across that day.

### Improved understanding of the role of device body placement

The CS-EMA component of the Mobile Teen app will collect information about how the smartphone is carried (e.g., in my pocket, in my bag, or purse). These data can assist researchers in understanding how activity level assessments using the smartphone’s built-in accelerometer may differ according to how or where the smartphone is worn on the body. Currently, there is some debate over optimal accelerometer placement (56, 57), and research is ongoing to determine the viability of detecting physical activities directly from mobile phone accelerometer data, regardless of how the phone is carried (58). The Mobile Team app will enable research into the viability of using the phone’s motion sensor in lieu of a separate objective monitor worn directly on the body.

### Improved understanding of upper body, load-bearing, and inclined activities

Data from the sensor-informed CS-EMA and end of the day components can be used to improve energy expenditure estimates for activities not well captured by waist-worn motion sensors such as those that involve the upper body, cycling, weight bearing, and incline or decline. Objective activity monitors worn on or near the waist (i.e., pocket) may not accurately measure activities that involve the upper body (e.g., hand cycle, rowing) (59). Waist-worn accelerometers may also not adequately capture cycling activities if the participant remains seated the entire time (60). Also, since objective activity monitors measure motion through acceleration, they often do not fully reflect true energy expenditure when the participant is load-bearing (e.g., heavy backpack, pushing a cart, carrying a child) or when motion involves uphill or downhill travel (61). After detected activity bouts (Rule 1), the CS-EMA component of the Mobile Teen app collects self-reported information about whether the activity involved cycling or upper body movements, load-bearing in terms of weight carried (e.g., *None, <5, 5–10 lbs*), and degree of incline involved (e.g., *mainly going uphill*, *mainly going downhill, mainly staying on flat ground*). These data can be used to upwardly or downwardly adjust energy expenditure estimates obtained from objective activity monitors.

### Improved understanding of activity type and purpose

The sensor-informed CS-EMA and end-of-day recall data from the Mobile Teen app may also be used to differentiate between conceptually distinct activity types (e.g., homework versus watching TV or soccer versus football), which may appear identical when examining objective activity intensity data alone. These distinctions are relevant in the context of behavior change interventions. For example, if the goal of an intervention is to decrease sedentary activity, it would be helpful to know what proportion of one’s sedentary activity is discretionary (e.g., TV watching, playing video games) as compared with non-discretionary (e.g., homework, required reading, practicing instruments) (22). This information is important to avoid possible unintended side effects of sedentary activity reduction interventions such as less time spent on homework. Also, the CS-EMA component gathers data about the purpose of the activity (e.g., *Fun/Recreation*, *To get somewhere*, *For work or housework*) that may be useful in assessing the amount of transit- and work-related physical activity performed.

### Improved understanding of contextual correlates of physical activity

The CS-EMA questions gather information about where, with whom, and why physical activity occurs; as well as how participants feel during those activities. These data help researchers to understand whether physical activity intensity or duration differs across contexts and to investigate time-varying antecedents and consequences of behavior. For example, using EMA mobile phone surveys, children’s moderate-to vigorous physical activity has been found to be greater outdoors than at home or at someone else’s house (21, 47). Also, engaging in more moderate-to-vigorous physical activity was associated with higher ratings of positive affect and feeling energetic, and lower ratings of negative affect in the subsequent 30 min (23). Theories of health behavior change could be enhanced by taking into account multilevel interactions between enduring person-level factors and moment-to-moment level fluctuations in contextual factors that may influence physical activity (62).

### Further testing

Further testing is planned that will compare the performance of the Mobile Teen app relative to that of the ACT in a free-living sample of *N* = 40 low-to-middle income, ethnically diverse adolescents in 9–12th grade. Subjects will be recruited through a Los Angeles area high school using informational flyers, posters, and classroom visits. To simplify the study administration and lower the study costs, we will only recruit adolescents who have a GSM-based mobile provider (AT&T or T-Mobile) so their personal phone SIM cards can be easily switched to temporary LG Nexus 4 smartphones with the Mobile Teen app installed for the duration of the study. Doing so will allow participants to use the study phone to make and receive calls and SMS messages with personal phone numbers. A within-person design will be used with two assessment conditions: (1) Mobile Teen app + ACT (MT + ACT) and (2) ACT, each administered for 14 days. The order of the assessment conditions (MT + ACT first versus ACT first) will be randomly assigned.

This comparison testing will evaluate the performance of the Mobile Teen app plus Actigraph (MT + ACT) versus ACT alone using three primary outcomes: (1) percentage of available activity data, (2) user satisfaction and comfort, and (3) research costs. ACT data collected during this testing will be flagged as *missing activity data* due to non-wear if the number of consecutive minutes with zero activity counts from the accelerometer is ≥60 (53). Software will be written to merge data from the ACT data with the data from the Mobile Teen app using internal time stamps generated by the devices. METs generated from the sensor-informed CS-EMA or end of the day recall components of the Mobile Teen app will be imputed where there is missing ACT data, and these episodes will be recoded as *available activity data*.

### Limitations

The Mobile Teen app has undergone iterative development and limited alpha and beta testing. Plans for more extensive testing with adolescents are underway, as described above. One possible concern with the method as proposed is that the Mobile Teen app depends upon adolescents in future activity measurement studies using personal mobile phones. Trends suggest (63–65), however, that within 5 years most adolescents in grades 9–12 will have phones with motion and location sensing. A related concern is that the phones they have will not be the appropriate phones for running the Mobile Teen app. In those cases, some of the adolescents could be switched to appropriate phones by temporarily swapping SIM cards, as proposed for the future Mobile Teen testing. The technology in its current form will only work on Android phones because iOS will not support the required background processing, but over 80% of new smartphone shipments use Android (66), and recent changes to Apple’s iPhone line adding a motion co-processor chip may allow continuous movement detection (67) and thereby create opportunities to develop versions of Mobile Teen for new iPhones as well.

As with all EMA, the interruption burden is high with the Mobile Teen app. Participants can theoretically be prompted more than once per hour, although in practice prompting is less frequent than that. However, our prior work (21) and ongoing pilot work with Mobile Teen suggests that high rates of compliance overall can be achieved with this technology. For example, in an EMA study using mobile phones, adults answered 82% of the surveys that were prompted (68). Another concern often raised with EMA is reactivity, the potential for behavior to be impacted by the very act of assessing it (69), but the magnitude of reaction to EMA has been observed to be small for EMA studies (70).

The Mobile Teen app records phone location in addition to accelerometer data, and the application does mark major location changes on the interface, as an additional memory cue. The location data may also be useful when chunking the data into bouts of specific types of behaviors, which we are exploring in current work. One open question is whether the phone can replace the need for other objective sensors entirely. If so, larger scale and longer term, but affordable, studies leveraging the phone technology adolescents will already have would become possible. This may be most feasible if the phone is worn in a consistent way on the body, such as in a holder on the hip, but because our pilot work will ascertain the location of the phone on the body, in a secondary analysis we will compare the quality of output of the Actigraph monitor and phone sensors in our study population.

### Conclusion and future directions

After testing is complete, the source code for Mobile Teen app will be made freely available to other researchers. This new software can be initially deployed in *combination* with other objective activity monitors, working side-by-side with standard activity monitors to improve compliance and quality of data collected. Eventually as smartphones with built-in motion and location sensors are validated for physical activity assessment, the adolescent’s own phones loaded with the Mobile Teen app can act as stand-alone activity measurement devices if adolescents will carry them in a standardized way. Overall, sensor-driven CS-EMA and end-of-day recall smartphone programs such as the Mobile Teen app have potential for deployment in large-scale epidemiological and intervention studies to improve the assessment of physical activity and sedentary behavior.

## Author Contributions

Genevieve Fridlund Dunton made substantial contributions to the conception and design of the work; and the acquisition, analysis, interpretation of data for the work. She also drafted the work and revised it critically for important intellectual content; gave final approval of the version to be published; and agreed to be accountable for all aspects of the work in ensuring that questions related to the accuracy or integrity of any part of the work are appropriately investigated and resolved. Eldin Dzubur made substantial contributions to the design of the work; and the interpretation of data for the work. He also drafted the work and revised it critically for important intellectual content; gave final approval of the version to be published; and agreed to be accountable for all aspects of the work in ensuring that questions related to the accuracy or integrity of any part of the work are appropriately investigated and resolved. Keito Kawabata made substantial contributions to the design of the work; and the acquisition of data for the work. He revised the work critically for important intellectual content; gave final approval of the version to be published; and agreed to be accountable for all aspects of the work in ensuring that questions related to the accuracy or integrity of any part of the work are appropriately investigated and resolved. Brenda Yanez made substantial contributions to the acquisition, analysis, interpretation of data for the work. She also drafted the work and revised it critically for important intellectual content; gave final approval of the version to be published; and agreed to be accountable for all aspects of the work in ensuring that questions related to the accuracy or integrity of any part of the work are appropriately investigated and resolved. Bin Bo made substantial contributions to the design of the work; and the acquisition of data for the work. He also revised the work critically for important intellectual content; gave final approval of the version to be published; and agreed to be accountable for all aspects of the work in ensuring that questions related to the accuracy or integrity of any part of the work are appropriately investigated and resolved. Stephen Intille made substantial contributions to the conception and design of the work; and the acquisition, analysis, interpretation of data for the work. He also drafted the work and revised it critically for important intellectual content; gave final approval of the version to be published; and agreed to be accountable for all aspects of the work in ensuring that questions related to the accuracy or integrity of any part of the work are appropriately investigated and resolved.

## Conflict of Interest Statement

The authors declare that the research was conducted in the absence of any commercial or financial relationships that could be construed as a potential conflict of interest.

## Footnotes

^1^The end-of-day activity labeling component of the app was described to participants as the “game” part of the application because it can be used to earn rewards if enough of the day is successfully labeled.

^2^This sampling strategy improves software reliability by permitting the software to run for only 20 s of each minute and then go into a lower-power state for the remaining 40 s. This permits the application to maintain a higher priority on the phone relative to others, thereby reducing the likelihood it will be shut down by app killer software attempting to improve battery life.

^3^Pilot testing raised concerns that some participants may select various *doing something else* options more often than the research team would like. Therefore, these options are excluded from inclusion in the Most Recent list.
